# Supplementary material for: Asymmetric Little–Parks oscillations in full shell double nanowires
Source: Sci Rep. 2021 Sep 24;11:19034. doi: 10.1038/s41598-021-97780-9 (PMC8463573; doi:10.1038/s41598-021-97780-9)
Supplement: Supplementary file 1 — Supplementary Information 1. [file 41598_2021_97780_MOESM1_ESM.pdf]

Supplementary Information: Asymmetric Little-Parks Oscillations in Full Shell Double Nanowires

CONTENTS

I. Little-Parks oscillations in 5 devices 2

II. Angular and perpendicular magnetic field dependence 3

III. Temperature dependence of Little-Parks oscillations 4

IV. Magnetic field remanence 6

References 6

I. LITTLE-PARKS OSCILLATIONS IN 5 DEVICES

Figure S1a-e shows data and fit of Little-Parks oscillations in five different devices. Parameters used for the fit can be found in Table S1 as well as their corresponding errors. The error bars are rough estimates changing only one parameter at a time, while keeping the other three fixed. All devices are fitted with the Little-Parks model with good agreement. Figures S1a,b correspond to Device 1 and 2 analyzed in the main text. To obtain a good fit, the parameter $A_\perp$ is chosen as a fitting parameter. In order to get an insight of the origin of $A_\perp$, we investigate this parameter versus junction length for the five devices shown in Fig. S1f. However, no correlation between the two parameters is found as expected [S1]. Figure S1g presents the correlation between the d^*/ξ ratio and the switching current at the first half-integer flux quantum $I_{\text{sw}}^{\Phi=\Phi_0/2}$. Here, d^* is the diameter of a cylinder with the same area as the two hexagonal nanowires (effective diameter) and ξ the coherence length extracted from the fits. It is observed that devices with small ratio exhibit destructive Little-Parks (Devices 2 and 3), while those with larger ratio exhibit non-destructive Little-Parks oscillations, in agreement with theoretical work [S2] and previous experiments in nanowires [S3, S4], even though our device cross section is ellipsoid and not circular.

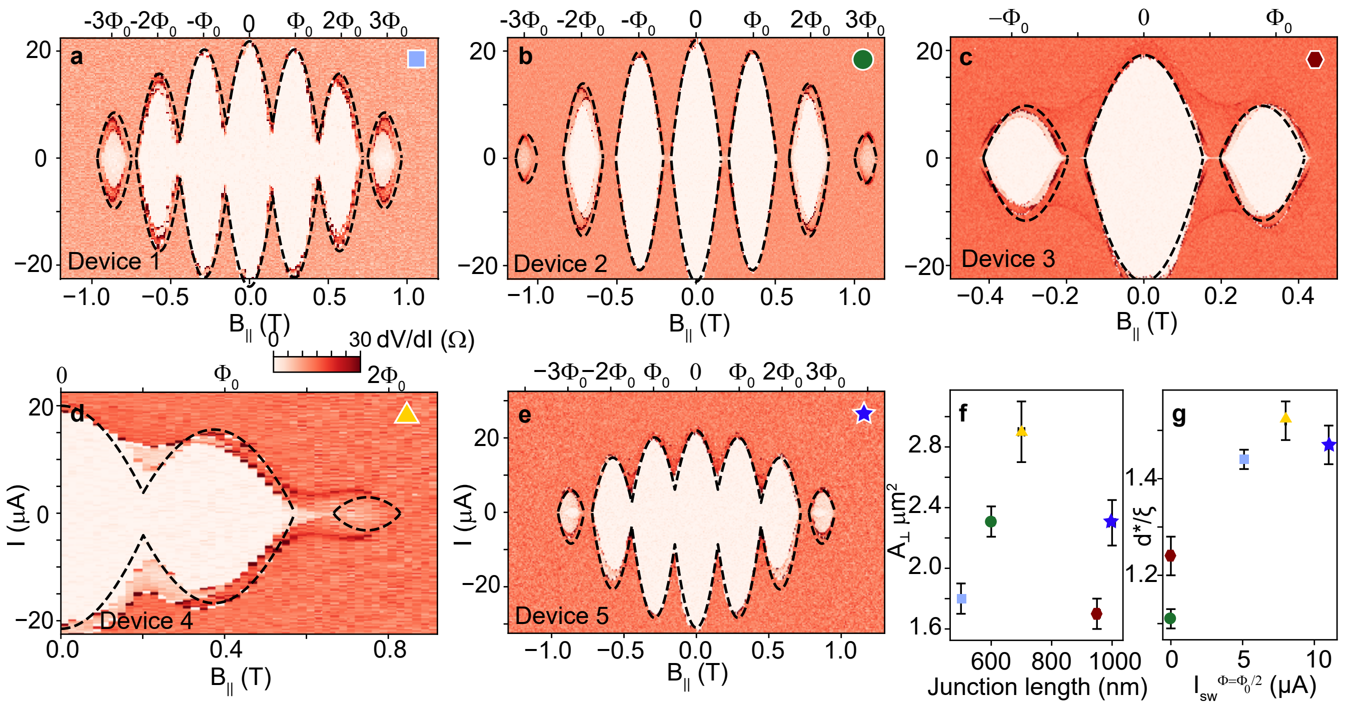

FIG. S1. **Little-Parks oscillations in five different nanowire devices.** (a,e) Differential resistance dV/dI measurements as a function of magnetic field B and applied current I for five different devices fabricated using five different double nanowires from the same growth batch. Dashed lines correspond to the fitted model. Panels b,c are the devices shown in the main text. (f) $A_\perp$ as a function of junction length of each device shown in panels (a-e). (g) d^*/ξ ratio as a function of I_{sw} at $\Phi = \Phi_0/2$.

TABLE S1. Model parameters used to fit data all devices in Fig. S1. From left to right, coherence length (ξ), effective perpendicular flux parameter ($A_\perp$), effective parallel flux area ($A_\parallel$), and ratio of shell thickness (t_s) to effective single cylinder diameter (d^*).

Device	ξ (nm)	$A_\perp$ (nm^2)	$A_\parallel$ (nm^2)	t_s/d^*
1	66 ±2	18000 ±1000	7100 ±50	0.13 ±0.05
2	81 ±2	23080 ±1000	5718 ±30	0.1 ±0.05
3	78 ±2	17000 ±700	6163 ±30	0.12 ±0.05
4	59 ±1	29000 ±2000	5187 ±40	0.12 ±0.05
5	65 ±2	23000 ±1000	7000 ±50	0.12 ±0.05

II. ANGULAR AND PERPENDICULAR MAGNETIC FIELD DEPENDENCE

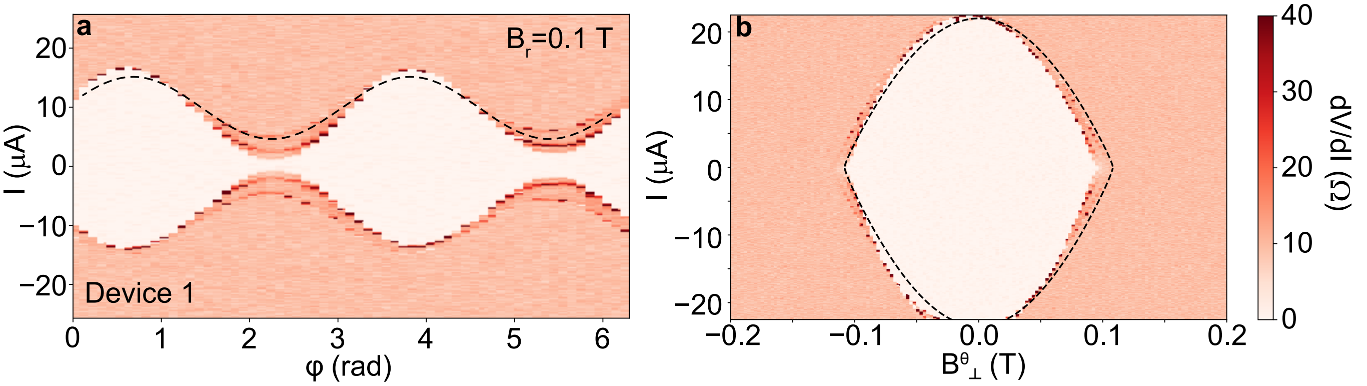

FIG. S2. **Dependence of critical current on field angle and perpendicular field** (a,b) Colormaps of differential conductance, dV/dI, plotted as a function of bias current, I, and (a) angle between the vector magnetic field $\mathbf{B}$ and coil X, φ, and (b) magnetic field applied perpendicular the double nanowires with misalignment $\theta = 4.4°$, $B_\perp^\theta$. In (a), the critical current shows clear modulations and it reaches its minimum (maximum) when ϕ is such that $\theta = 90$ ($\theta = 0$), reflecting the phenomenological finding that the area threaded by parallel magnetic flux is smaller than the effective perpendicular parameter ($A_\parallel < A_\perp$). The critical perpendicular field found from the measurement in (b) is significantly smaller than the parallel critical field (0.1 T against 0.95 T), supporting the data and interpretation in (a). The asymmetry against $\pm B_\perp^\theta$ ($\varphi = 2.2$, 5.34 rad) in both (a) and (b) is attributed to coil remanence. The data was fitted with a calculation of the critical current, I_c (dashed lines), using the single hollow superconducting cylinder model. Reasonably good fits are obtained, with the fit quality decreased due to asymmetries. Data obtained from Device 1.

Here we analyze the dependence of I_c as a function of a rotating magnetic field of magnitude $B_r = 0.1$T, showing that the two parameters $A_\perp$ and $A_\parallel$ defined in the main text have to be different in order to have a modulating critical current versus a rotating magnetic field. Figure S2a shows such a measurement from Device 1, where the black dashed-line is the model we use based on Eqs. 1,2 of the main text. As the applied magnetic field is weak, we have $n = 0$ and the last term of Eq. 2 ($\alpha_\parallel$) is negligible. As a result, $\alpha_\parallel$ and $\alpha_\perp$ would be identical if the two parameters are the same. A calculation from Ref. [S1] for a solid cylinder found a factor of 2 between the parameters $A_\parallel$ and $A_\perp$ ($A_\perp/A_\parallel$=2), while we experimentally find a factor of $A_\perp/A_\parallel \approx 2.5 - 5$ for our elliptical hollow cross-sectional (13 nm shell) full-shell nanowires agreeing with the calculation on $A_\perp$ being larger than $A_\parallel$. Theoretical work on our specific system is

needed to understand if these findings are consistent with theory.

Figure S2b presents an $I - B$ measurement of Device 1 as a function of dV/dI at an angle perpendicular to nanowire axis.The black dashed-line is the fit, showing good agreement to the data, using the same parameters as in Fig. S2a and in Fig. 2 of the main text.

III. TEMPERATURE DEPENDENCE OF LITTLE-PARKS OSCILLATIONS

Figure S3 presents Little-Parks oscillations of I_c and T_c of Device 3 to justify Eq. 4 of the main text. The relation between the zero-field and finite-field critical current and critical temperature noted in Eq. 4 of the main text is verified in the data. Explicitly,

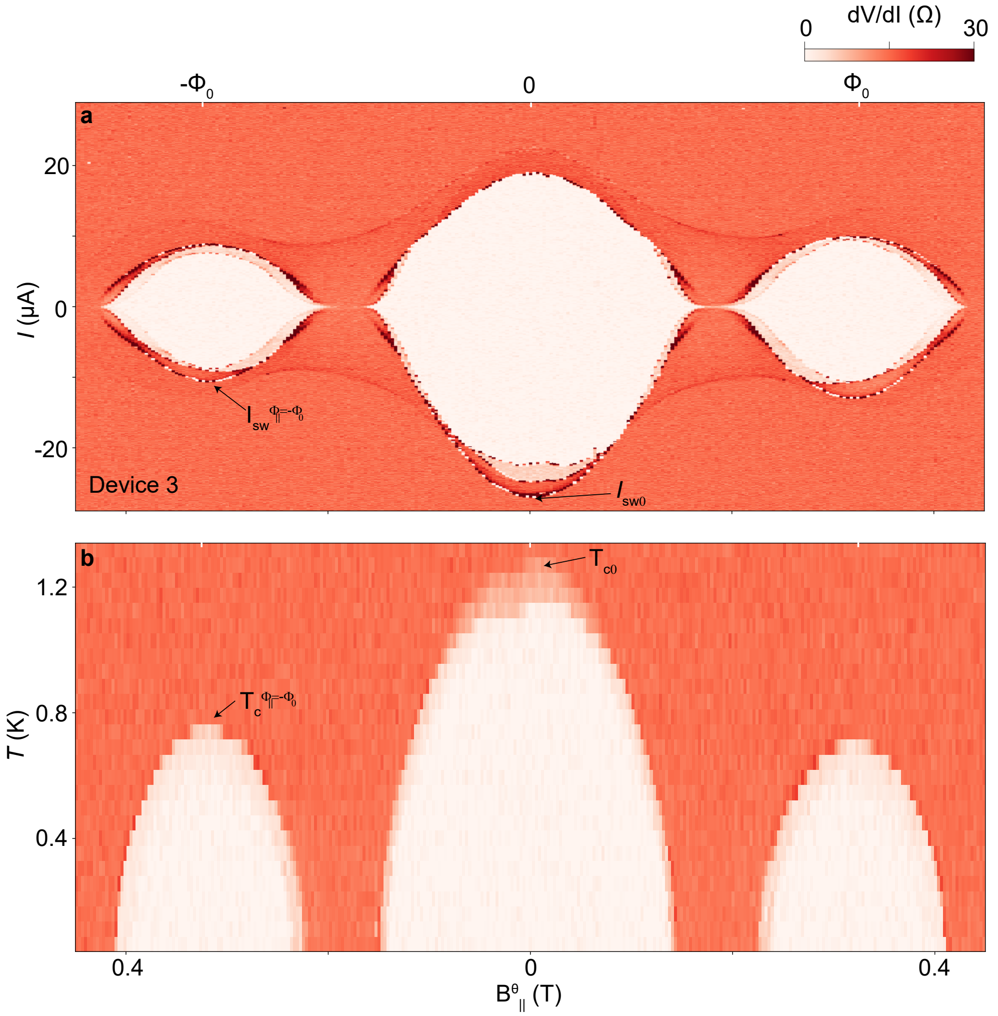

FIG. S3. **Relation of I_c to T_c Little-Parks oscillations.** (a,b) Colormaps of differential conductance, dV/dI, plotted as a function of magnetic field applied parallel to the double nanowires with misalignment $\theta = 10.7°$, $B_{\parallel}^{\theta}$, and (a) bias current, I swept from positive to negative values, at $T = 30$ mK and (b) refrigerator temperature, T, at $I = 0.7$ μA. Little-Parks oscillations of I_c in (a) correlate to oscillations of T_c in (b), supporting our use of Eq. 4 in the main text. Data obtained from Device 3.

at $B_{\parallel}^{\theta} = 0$ we measure $I_{\text{sw}}(B_{\parallel}^{\theta} = 0) = -24\ \mu$A and $T_{\text{c}}(B_{\parallel}^{\theta} = 0) = 1.3$ K, and at $B_{\parallel}^{\theta}$ such that $\frac{\Phi_{\parallel}}{\Phi_0} = -1$ we measure $I_{\text{sw}}(\frac{\Phi_{\parallel}}{\Phi_0} = -1) = -10\ \mu$A and $T_{\text{c}}(\frac{\Phi_{\parallel}}{\Phi_0} = -1) = 0.75$ K. Negative values of current are chosen to capture the switching current as the sweep direction is from positive to negative. For these values we verify the relation between $I_{\text{c}}/I_{\text{c0}}$ and $T_{\text{c}}/T_{\text{c0}}$ in Eq. 4 of the main text within 5%, assuming that $I_{\text{sw}} = I_{\text{c}}$. The lack of additional lobes beyond the ones at $\frac{\Phi_{\parallel}}{\Phi_0} = \pm 1$ is due to a large angle misalignment deduced from the fit in Fig. S1d, which effectively reduces the critical magnetic field of the device.

IV. MAGNETIC FIELD REMANENCE

In this section we discuss the finite magnetic field remanence from the X and Z coils that were used to probe the Little-Parks effect in our devices. Figures S4a,b show measurements of I_c as a function of B_x swept upwards and downwards respectively, as noted by the red arrow. Note that the maximum value of I_c shifts by $\approx$ 10 mT between the two panels. Figures S4c,d exhibit the same effect for the Z coil B_z. This effect explains the small inconsistencies - which are mostly visible near zero magnetic field - between datasets, when the magnetic field sweep direction changes.

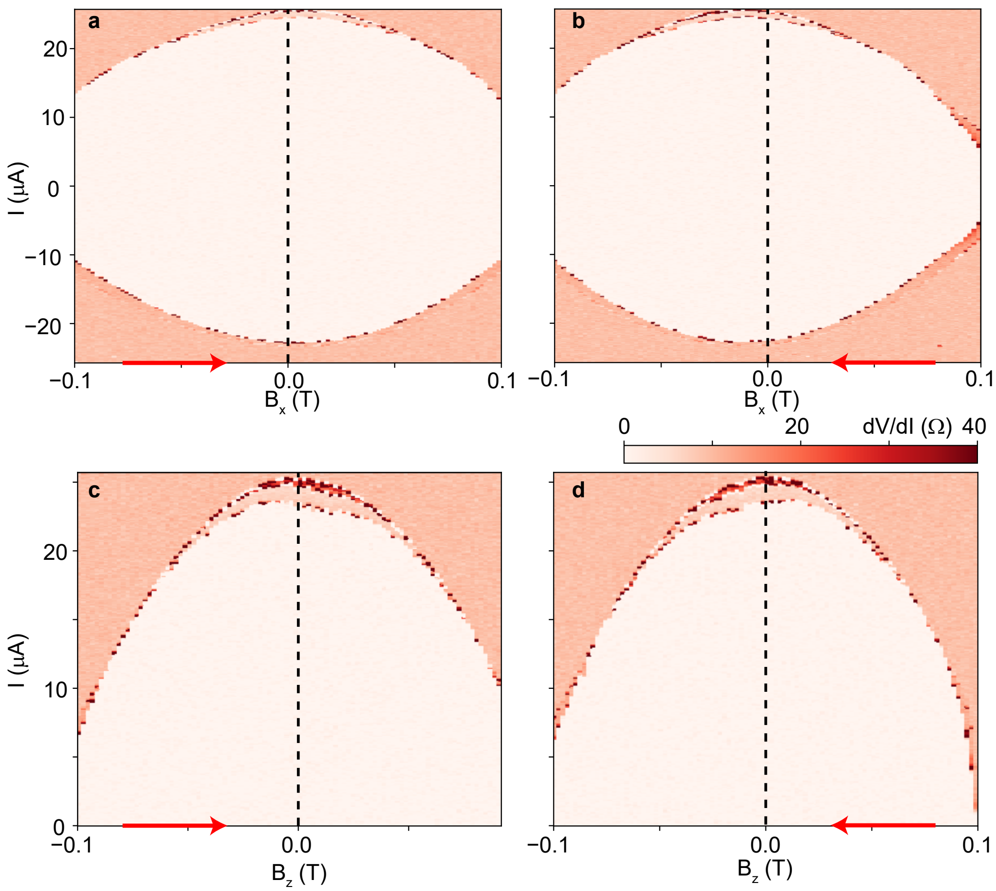

FIG. S4. **Evidence for finite remanence in coils X and Z.** (a,b) Colormaps of differential conductance, dV/dI, plotted as a function of field in (a,b) coil X, B_x and (c,d) coil Z, B_z and positive bias current, I. Arrows on the horizontal axis indicate field direciton. The pattern of destruction of superconductivity is asymmetric and hysteretic on the direction of B_x and B_z, with a field-direction dependent small offset of < 20 mT seen in both cases.

[S1] N. Shah and A. Lopatin, Phys. Rev. B **76**, 094511 (2007).
[S2] G. Schwiete and Y. Oreg, Physical review letters **103**, 037001 (2009).
[S3] S. Vaitiekėnas, G. Winkler, B. van Heck, T. Karzig, M.-T. Deng, K. Flensberg, L. Glazman, C. Nayak, P. Krogstrup, R. Lutchyn, *et al.*, Science **367** (2020).
[S4] S. Vaitiekėnas, P. Krogstrup, and C. Marcus, Physical Review B **101**, 060507 (2020).
